# The serine protease homolog CLIPA14 modulates the intensity of the immune response in the mosquito *Anopheles gambiae*

**DOI:** 10.1074/jbc.M117.797787

**Published:** 2017-09-19

**Authors:** Johnny Nakhleh, George K. Christophides, Mike A. Osta

**Affiliations:** From the ‡ Department of Biology, American University of Beirut, Beirut 1107 2020, Lebanon and; § Department of Life Sciences, Imperial College London, London SW7 2AZ, United Kingdom

**Keywords:** host defense, host-pathogen interaction, immunology, malaria, plasmodium, clip domain serine protease, melanization, mosquito immunity

## Abstract

Clip domain serine protease homologs (SPHs) are positive and negative regulators of *Anopheles gambiae* immune responses mediated by the complement-like protein TEP1 against *Plasmodium* malaria parasites and other microbial infections. We have previously reported that the SPH CLIPA2 is a negative regulator of the TEP1-mediated response by showing that CLIPA2 knockdown (kd) enhances mosquito resistance to infections with fungi, bacteria, and *Plasmodium* parasites. Here, we identify another SPH, CLIPA14, as a novel regulator of mosquito immunity. We found that CLIPA14 is a hemolymph protein that is rapidly cleaved following a systemic infection. *CLIPA14* kd mosquitoes elicited a potent melanization response against *Plasmodium berghei* ookinetes and exhibited significantly increased resistance to *Plasmodium* infections as well as to systemic and oral bacterial infections. The activity of the enzyme phenoloxidase, which initiates melanin biosynthesis, dramatically increased in the hemolymph of *CLIPA14* kd mosquitoes in response to systemic bacterial infections. Ookinete melanization and hemolymph phenoloxidase activity were further increased after cosilencing *CLIPA14* and *CLIPA2*, suggesting that these two SPHs act in concert to control the melanization response. Interestingly, *CLIPA14* RNAi phenotypes and its infection-induced cleavage were abolished in a TEP1 loss-of-function background. Our results suggest that a complex network of SPHs functions downstream of TEP1 to regulate the melanization reaction.

## Introduction

Clip domain serine proteases (CLIPs)^2^ are key components of insect immune responses leading to melanization and antimicrobial peptide synthesis through the Toll pathway. CLIPs are specific to invertebrates and form large gene families in insect genomes (1). Their function has been particularly studied in the context of the melanization response, which, in insects, plays important roles in several physiological processes, including cuticle sclerotization or tanning (2), hardening of wound clots (3), and resistance to microbial infections (4–7). There is also convincing evidence for an antiviral role of melanization (8, 9). The infection-induced melanotic response is initiated when pattern recognition receptors (PRRs) bind to microbial cell components, triggering the activation of a cascade of serine proteases, constituted mostly of CLIPs, which culminates in the limited proteolytic cleavage of the zymogen prophenoloxidase (PPO) into active phenoloxidase (PO), the rate-limiting enzyme in melanin biosynthetic pathways.

The serine protease cascades acting upstream of PPO are complex and finely regulated to control the spatial, temporal, and intensity of PPO activation (for reviews, see Refs. 10 and 11). The initiator protease in these cascades is a modular serine protease composed of a complex assortment of domains that allow multiple interactions with upstream PRRs and downstream proteases (12–14). The serine proteases acting downstream of modular serine protease are CLIPs. Among these are non-catalytic CLIPs (also called clip domain serine protease homologs (SPHs)), which lack one or more of the three residues (His, Asp, and Ser) that form the protease catalytic triad. Both catalytic CLIPs and SPHs are secreted as precursor proteins and require cleavage at a specific site between the clip and protease domains to become active. The role of SPHs as inferred from studies in other insects seems to be confined to the terminal step of PPO cleavage (for a review, see Ref. 10). SPHs act as cofactors for terminal CLIPs in the cascade, called prophenoloxidase-activating proteases (PAPs), for the efficient cleavage and activation of PPO (15–17). In general, PPOs cleaved by PAPs in the absence of SPH cofactors showed no activity *in vitro* even when the SPH was added later to the active PO (15, 18, 19), indicating that SPHs are required for correct cleavage of PPOs. However, in one study, *Anopheles gambiae* CLIPB9 was shown to cleave *Manduca sexta* PPO in the absence of SPH, generating catalytically active PO, suggesting that in certain instances SPHs may not be required for PPO activation (20).

Studies in the malaria vector *A. gambiae* revealed that SPHs have a broader role in the regulation of immune responses. A systematic functional genetic screen by RNA interference (RNAi) identified several SPHs (CLIPA8, CLIPA2, CLIPA5, and CLIPA7) to be involved in the melanization of the rodent malaria parasite *Plasmodium berghei* while invading the mosquito midgut epithelium (21). CLIPA8 acts as a positive regulator of the melanization response triggered against bacterial (22) and fungal infections (7) as well as against infections with *P. berghei* in certain mosquito melanotic backgrounds (21). CLIPA8 is cleaved following bacterial challenge, and this cleavage is controlled by thioester-containing protein 1 (TEP1) (23), a homolog of the mammalian C3 complement factor that mediates key effector functions in mosquito immune responses, including microbial lysis, phagocytosis, and melanization (24–28). The knockdown of either *TEP1* or *CLIPA8* abolished hemolymph PO activity in response to bacterial infections (7, 23), indicating a tight control by TEP1 over the melanization response.

The RNAi phenotypes of CLIPA2, CLIPA5, and CLIPA7 suggested a negative regulatory role for these SPHs in the melanization response to *P. berghei* (21). Recently, CLIPA2 was shown to regulate melanization indirectly by controlling TEP1 activity during systemic infections; *CLIPA2* kd enhanced TEP1 activity, leading to an exaggerated PO activity in the hemolymph following *Escherichia coli* infections (29, 30). CLIPA2 is thought to negatively regulate the conversion of full-length TEP1 (TEP1-F) to the processed form (TEP1_cut_), which was shown to be the active form of TEP1 that is stabilized by the two leucine-rich immune proteins APL1C and LRIM1 (31, 32). A more recent study identified the SPH SPCLIP1 as a major positive regulator of TEP1 whereby the localization of TEP1 and SPCLIP1 to *Plasmodium* ookinetes was shown to be mutually dependent (23).

Here, we show that a novel SPH termed CLIPA14 acts as a major negative regulator of the mosquito melanization response, acting downstream of TEP1 and SPCLIP1. We have previously shown that CLIPA14 coimmunoprecipitates with CLIPA2 from mosquito hemolymph extracts (29). RNAi-mediated silencing of *CLIPA14* in adult female *A. gambiae* mosquitoes triggered melanization of most *P. berghei* ookinetes invading their midgut in a TEP1-dependent manner. These mosquitoes exhibited an unusually high hemolymph PO activity following bacterial systemic infections in addition to strong resistance to systemic and oral bacterial infections. We also show that the melanization of ookinetes and hemolymph PO activity were significantly enhanced when *CLIPA14* and *CLIPA2* were cosilenced, suggesting that they act in concert to regulate the TEP1-mediated melanization response. Our results reveal a new level of complexity in SPH function in mosquito immunity and provide further evidence for their key role in regulating the mosquito complement-like response.

## Results

### CLIPA14 regulates Plasmodium melanization in a TEP1-dependent manner

**Figure 1.**
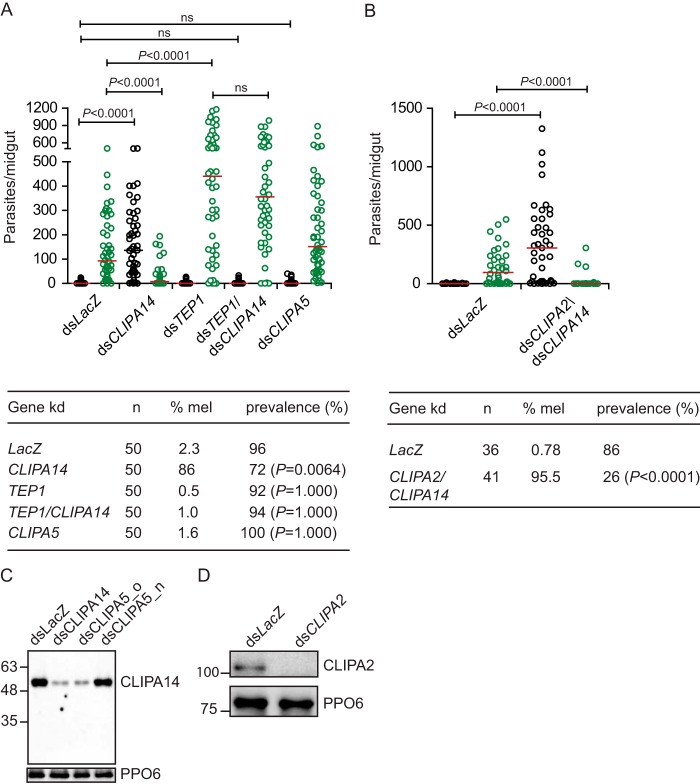
***CLIPA14* kd triggers a potent TEP1-dependent melanotic response against malaria parasites.** *A* and *B*, scatter plots of live GFP-expressing *P. berghei* oocysts (*green circles*) and dead melanized (*mel*) ookinetes (*black circles*) scored in the midguts of the indicated mosquito genotypes 7 days postinfection. *Red lines* indicate median parasite numbers. Statistical analysis for the parasite distribution was performed using the Mann–Whitney log-rank test, and *p* values less than 0.05 were considered significant. Data were pooled from three independent biological experiments. Statistical analysis for prevalence was performed using the χ^2^ test followed by Fisher's exact test, and *p* values less than 0.05 were considered significant. *C*, Western blot measuring the efficiency of *CLIPA14* silencing in hemolymph extracts of mosquitoes treated with the indicated dsRNAs. The blot was probed with PPO6 antibody to confirm equal loading. Note that the old dsRNA (ds*CLIPA5*_*o*) we used previously to silence *CLIPA5* (21) cross-silenced *CLIPA14* with the same efficiency as ds*CLIPA14*, whereas the new CLIPA5-specific dsRNA (ds*CLIPA5*_*n*) did not. *D*, Western blot showing CLIPA2 silencing in the hemolymph of naïve mosquitoes at day 4 after ds*CLIPA2* injection. *ns*, non-significant.

We have previously identified CLIPA14 among the list of proteins that coimmunoprecipitated with CLIPA2 in hemolymph extracts of *Beauveria bassiana-*infected mosquitoes (29). To address the function of CLIPA14 in mosquito immunity, we first silenced this gene in adult female *A. gambiae* mosquitoes by RNAi and scored the effect on the survival of *P. berghei* oocysts at day 7 after ingestion of an infectious blood meal. Interestingly, *CLIPA14* kd triggered a potent melanization response against ookinetes, resulting in the melanotic encapsulation of the majority (86%) of these parasite stages (Fig. 1*A*); in 26% of dissected mosquitoes, midguts contained only melanized ookinetes with no single live oocyst detected. *LacZ* kd controls revealed a background level of melanization as expected for the G3 strain. This *CLIPA14* RNAi phenotype is stronger than that observed for *CLIPA2* (21, 30) and similar to that previously reported for *CTL4* kd mosquitoes (33). Parasite melanization was abolished when *CLIPA14* and *TEP1* were cosilenced, and oocyst counts were similar to those in *TEP1* single kd mosquitoes, indicating that the enhanced immunity against parasites is TEP1-dependent. This result confirms the central role of TEP1 in initiating the melanization response as reported previously in different mosquito genetic backgrounds (24, 30, 34).

Phylogenetic analysis of *A. gambiae* CLIPAs revealed that CLIPA5 is the closest paralog to CLIPA14 (1). However, when we aligned the protein sequences of CLIPA5 and CLIPA14 (AGAP011788) available in VectorBase (www.vectorbase.org)^3^ using the MUSCLE alignment tool, we found that the CLIPA14 sequence is partial and is missing ∼146 amino acids from the C terminus of its protease-like domain (supplemental Fig. S1). We reconstructed the full-length *CLIPA14* cDNA using the GENSCAN web server at Massachusetts Institute of Technology (genes.mit.edu/GENSCAN.html)^3^ (35) to determine coding sequences downstream (3′ flanking) of the *CLIPA14* cDNA available in VectorBase. We designed primers based on the newly predicted sequence, amplified full-length CLIPA14 from *A. gambiae* G3 cDNA, and cloned it into pIEx-10 expression plasmid. The sequence of the cloned amplicon matched the predicted sequence we reconstructed using GENSCAN, indicating that CLIPA14 has a gene structure typical of a clip domain-containing SPH. However, we found that this gene segment of CLIPA14 that was missing from the annotation contained a contiguous sequence of 53 nucleotides that showed 100% complementarity to a region of the double-stranded RNA (dsRNA) we used previously to silence CLIPA5 (supplemental Fig. S2) in the context of a large genetic screen of *A. gambiae* CLIPs (21). It is worth noting that, at that time, CLIPA14 was absent from the initial list of *A. gambiae* annotated genes based on which the CLIP gene screen was performed (36). This raised the possibility that the CLIPA5 RNAi phenotype characterized by increased ookinete melanization (21) could be due to cross-silencing CLIPA14. To address this issue, we resilenced CLIPA5 with a new gene-specific dsRNA complementary to the region situated between the clip domain and the beginning of the protease domain; this region shows significant sequence diversification in CLIPA14 and CLIPA5, which exhibit their highest sequence similarity in their protease domains, especially in the last 241 amino acids (supplemental Fig. S1). Following infection by *P. berghei*, *CLIPA5* kd mosquitoes exhibited a basal level of ookinete melanization similar to that in *LacZ* kd controls (Fig. 1*A*), indicating that CLIPA5 does not regulate melanization and that its previous RNAi phenotype is due to cross-silencing CLIPA14. Indeed, Western blot analysis revealed that CLIPA5 dsRNA used in our previous genetic screen (21) silenced *CLIPA14* with a similar efficiency as ds*CLIPA14* did (Fig. 1*C*). Despite the significant sequence similarity between CLIPA14 and CLIPA5, CLIPA14 antibody does not cross-react with CLIPA5 because CLIPA14 signal in *CLIPA14*/*CLIPA5* double kd (dkd) was similar to that in *CLIPA14* single kd mosquitoes (supplemental Fig. S3B). It is worth noting that *CLIPA14* kd strongly reduces CLIPA14 hemolymph levels but does not deplete it as reported for other *CLIPA* kd (22, 30); however, this reduction is sufficient to observe the phenotype.

*CLIPA2* kd mosquitoes also melanize a significant number of ookinetes in a TEP1-dependent manner (30). The RNAi phenotypes of CLIPA2 and CLIPA14 suggest that they play non-redundant roles in fine-tuning the melanization response. Hence, we asked whether a higher level of parasite melanization can be achieved if both genes are cosilenced. Interestingly, most *CLIPA14*/*CLIPA2* dkd mosquitoes were completely refractory to *P. berghei*; the infection prevalence (*i.e.* percentage of mosquitoes carrying live parasites) was 26% (Fig. 1*B*) as compared with 72 and 76% for *CLIPA14* (Fig. 1*A*) and *CLIPA2* (30) single knockdowns, respectively. Western blot analysis showed that *CLIPA2* was efficiently silenced by RNAi (Fig. 1*D*) as reported previously (30). This enhanced melanotic response is not due to cross-silencing between *CLIPA2* and *CLIPA14* nor is it due to the compromised secretion of CLIPA14 in *CLIPA2* kd (supplemental Fig. S3A). Indeed, real-time PCR analysis revealed that ds*CLIPA14* specifically silenced its target gene without affecting *CLIPA2* or *CLIPA5* expression levels (supplemental Fig. S3C). These results indicate that CLIPA2 and CLIPA14 exhibit additive roles in the mosquito melanization response.

### CLIPA14 kd mosquitoes are resistant to bacterial infections

**Figure 2.**
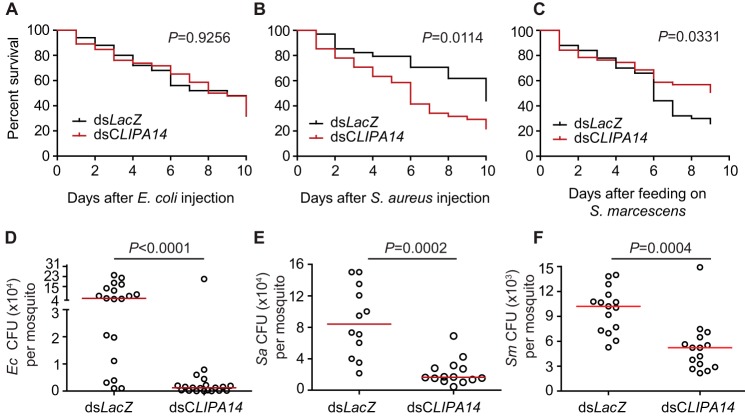
***CLIPA14* kd increases resistance to systemic and oral bacterial infections.** *CLIPA14* kd mosquitoes are resistant to bacterial infections. *A–C*, mosquito survival assays following injection of *E. coli* (*A*_600 nm_ = 0.4) (*A*) and *S. aureus* (*A*_600 nm_ = 0.4) (*B*) into mosquito hemocoel or after oral infection with *S. marcescens* (C). One representative experiment is shown from three independent biological experiments. The two other replicates are shown in supplemental Fig. S4. The Kaplan–Meier survival test was used to calculate percent survival. Statistical significance of the observed differences was calculated using the log-rank test. *D–F*, bacterial proliferation assays conducted on mosquitoes injected with *E. coli* (*Ec*) (*A*_600 nm_ = 0.4) (*D*) and *S. aureus* (*Sa*) (*A*_600 nm_ = 0.4) (*E*) or fed on sugar pads containing *S. marcescens* (*Sm*) (*A*_600 nm_ = 0.1) (*F*). Batches of eight whole mosquitoes (*D* and *E*) or mosquito carcasses (excluding midgut) (*F*) were ground in LB medium at 48 h after infection, and cfu were scored on LB plates supplemented with the appropriate antibiotic. Each point on the scatter plot represents the mean cfu per mosquito per batch. Statistical analysis was performed using the Mann–Whitney log-rank test. Medians (*red lines*) were considered significant if *p* was <0.05. Data shown are from three independent biological experiments.

The potent melanotic response elicited by *CLIPA14* kd mosquitoes against *Plasmodium* ookinetes does not necessarily indicate that these mosquitoes are similarly resistant to infections with other classes of microorganisms. This is supported by the RNAi phenotypes previously reported for the *A. gambiae CTL4* gene. Although *CTL4* kd mosquitoes exhibited a similar level of resistance to *Plasmodium* ookinetes as *CLIPA14* mosquitoes (33), they were more susceptible to systemic *E. coli* infections (37). To address the role of CLIPA14 in antimicrobial defense, *CLIPA14* kd mosquitoes were challenged with bacteria either by direct injection into the hemocoel or through the oral route. *LacZ* kd mosquitoes were used as controls. The results showed that *CLIPA14* kd mosquitoes harbored significantly less *E. coli* (Fig. 2*D*) and *Staphylococcus aureus* (Fig. 2*E*) in their tissues at 48 h after bacterial injections as compared with controls. Interestingly, although the survival rates of *E. coli*-infected *CLIPA14* kd mosquitoes were similar to controls (Fig. 2*A* and supplemental Fig. S4A), *S. aureus*-infected *CLIPA14* kd mosquitoes showed compromised survival compared with controls (Fig. 2*B* and supplemental Fig. S4B) despite harboring fewer bacteria. Indeed, two criteria are known to influence the host survival to infections: reducing the microbial burden in tissues and tolerating the damage triggered by the immune response and the microbe (38, 39). Hence, the compromised survival of *CLIPA14* kd mosquitoes to *S. aureus* but not to *E. coli* infections could be due to an increased magnitude of the immune response in the presence of the former bacteria, possibly leading to tissue pathology and host toxicity. In contrast, *CLIPA14* kd in naïve mosquitoes did not influence mosquito survival (supplemental Fig. S5).

The direct injection of bacteria into the hemocoel has been widely used as a practical approach to trigger systemic infections in mosquitoes and other insects despite that this route of infection is unlikely to be common in natural habitats due to the rigidity of the external cuticle. So we examined whether *CLIPA14* kd mosquitoes are also able to clear oral infections with *Serratia marcescens* bacteria that are commonly present within the gut flora of field-caught mosquitoes (40–42) and known to invade the insect gut epithelium, reaching into the hemocoel (43). Silencing *CLIPA14* resulted in significantly lower numbers of *S. marcescens* in the hemocoel compared with *LacZ* kd controls (Fig. 2*F*), indicating that CLIPA14 modulates the mosquito immune response to oral bacterial infections. Also, *CLIPA14* kd mosquitoes exhibited enhanced tolerance to *S. marcescens* infections (Fig. 2*C* and supplemental Fig. S4C), suggesting that Gram-negative and Gram-positive bacteria differentially influence host tolerance in this genetic background.

**Figure 3.**
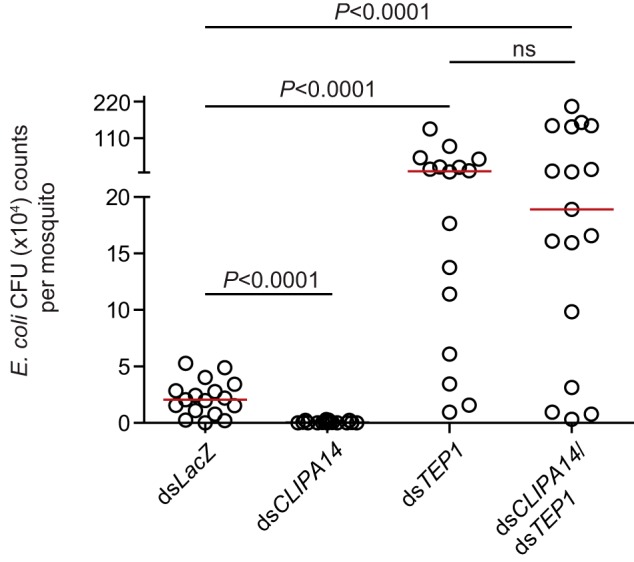
**The resistance of *CLIPA14* kd mosquitoes to bacterial infections is TEP1-dependent.** Bacterial cfu were scored in the indicated mosquito genotypes at 48 h after injection with *E. coli* (*A*_600 nm_ = 0.4). For each genotype, batches of eight whole mosquitoes were ground in LB medium, and cfu were scored on LB plates supplemented with ampicillin. Each point on the scatter plot represents the mean cfu per mosquito per batch. Statistical analysis was performed using the Mann–Whitney log-rank test. Medians (*red lines*) were considered significant if *p* was <0.05. Data shown are from three independent biological experiments. *ns*, non-significant.

The enhanced bacterial clearance in *CLIPA14* kd mosquitoes prompted us to address the contribution of TEP1, which is known to play an important role in antibacterial defense (26, 29). To address this point, *E. coli* colony-forming units (cfu) in *LacZ* (control), *CLIPA14*, and *TEP1* single kd were compared with those in *CLIPA14*/*TEP1* dkd mosquitoes at 48 h after bacterial injection into the hemocoel. As expected, *TEP1* kd triggered increased proliferation of *E. coli* compared with the control group (Fig. 3). Interestingly, *E. coli* cfu in the dkd group were similar to those in the *TEP1* single kd, clearly indicating that rapid bacterial clearance in *CLIPA14* kd mosquitoes is TEP1-dependent.

### CLIPA14 is cleaved in response to bacterial systemic infections

**Figure 4.**
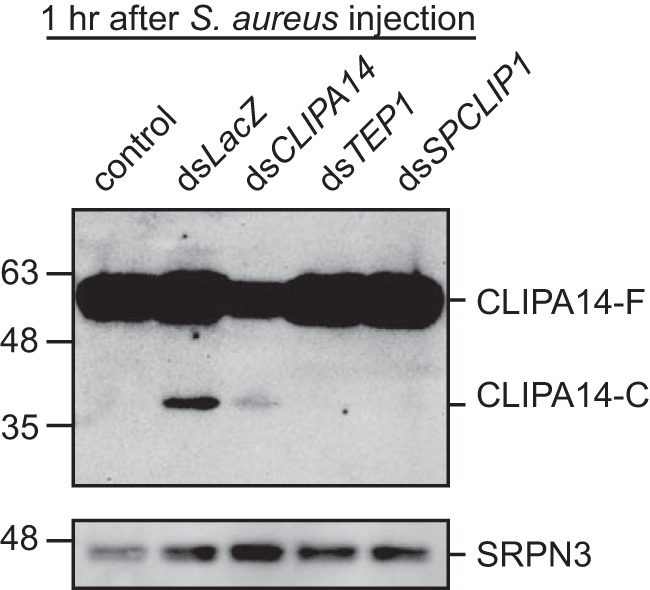
**TEP1 and SPCLIP1 control the infection-induced cleavage of CLIPA14.** The Western blot shows CLIPA14 cleavage in the indicated mosquito genotypes. Hemolymph was extracted from mosquitoes at 1 h after injection with *S. aureus* (*A*_600 nm_ = 0.8). Protein quantification was performed using the Bradford protein assay, and 1.2 μg of hemolymph proteins were loaded per lane. The membrane was probed with antibodies against CLIPA14 and SRPN3 (as a loading control). Shown is a high exposure image (saturated) to detect the cleaved CLIPA14-C. The image is representative of three independent biological experiments.

SPHs require proteolytic cleavage by catalytic CLIPs to become functional despite their lack of catalytic activity. This has been shown in several insect SPHs, including SPH1 and SPH2 of *M. sexta* (44, 45), PPAFII (proPO activating factor-II) of *Holotrichia diomphalia* (16), and CLIPA8 of *A. gambiae* (22). CLIPA14 does not seem to be an exception in this regard because injecting mosquitoes with *S. aureus* triggered its rapid cleavage in the hemolymph (Fig. 4 and supplemental Fig. S6A). CLIPA14 was also cleaved after *E. coli* injections but sometimes at a weaker level (data not shown), which prompted the use of *S. aureus* in these assays. The apparent molecular masses of full-length CLIPA14 (CLIPA14-F) and the cleaved C-terminal domain (CLIPA14-C) are 59 and 39 kDa, respectively. We were not able to detect the N-terminal fragment containing the clip domain, possibly because it is weakly antigenic compared with the larger C-terminal protease-like domain, hence generating an undetectable chemiluminescence signal. Of note, even CLIPA14-C band was always weak and required high exposures (signal saturation) for clear detection, suggesting that either a small fraction of CLIPA14-F is cleaved or the cleaved product is quickly sequestered, possibly on microbial surfaces. *CLIPA14* kd strongly reduced CLIPA14 hemolymph levels by ∼85% (supplemental Fig. S6B); this reduction is not apparent in Fig. 4 as it is overexposed. Interestingly, silencing either TEP1 or its positive regulator SPCLIP1 completely abolished CLIPA14 cleavage (Fig. 4 and supplemental Fig. S6A), indicating that CLIPA14 is tightly controlled by the TEP1 pathway.

### CLIPA14 controls the level of hemolymph PPO activation during systemic infections

**Figure 5.**
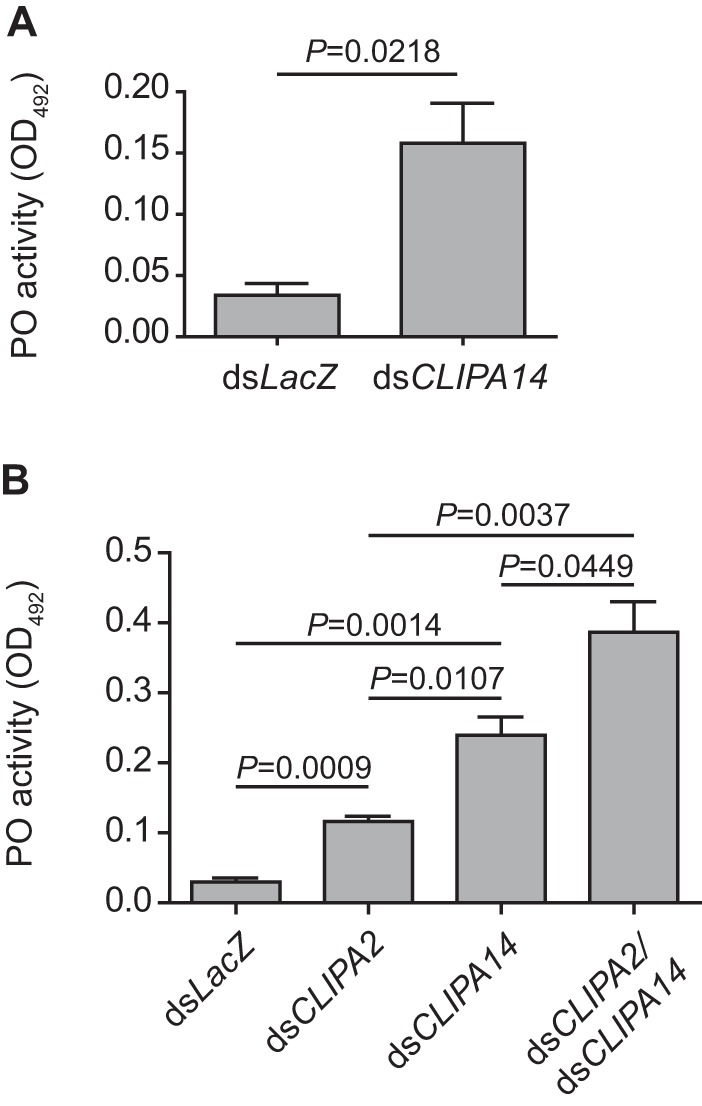
**CLIPA14 regulates the intensity of hemolymph PPO activation.** PO enzymatic activity (detected as absorbance at *A*_492 nm_ after conversion of l-DOPA) was measured in hemolymph extracted from ds*LacZ* (control), ds*CLIPA2*, ds*CLIPA14*, and ds*CLIPA2/*ds*CLIPA14* mosquitoes at 3 h postinjection of live *E. coli* (*A*_600 nm_ = 0.8). The graphs show PO activity measured at 30 min after addition of l-DOPA. Means were calculated from three independent biological experiments. *Error bars* represent standard deviation of the mean. Statistical analysis was performed using the Student's *t* test, and differences were considered to be significant if *p* was <0.05.

The fact that *CLIPA14* kd triggered a potent melanotic response against *Plasmodium* ookinetes prompted us to ask whether these mosquitoes elicit an enhanced PO activity against systemic bacterial infections. To address this point, *CLIPA14* and *LacZ* kd mosquitoes were injected with a suspension of live *E. coli* (*A*_600 nm_ = 0.8), and hemolymph was extracted 3 h later to measure PO activity. Our data revealed an ∼4.5 times higher PO activity in *CLIPA14* compared with that in the infected *LacZ* kd control (Fig. 5*A*), indeed indicating the presence of an enhanced melanotic response. *CLIPA2* kd mosquitoes were also shown previously to exhibit enhanced hemolymph PO activity following *E. coli* infections (30). Hence, we compared the hemolymph PO activities in both genotypes to determine which of these SPHs is a more potent regulator of the systemic melanization response. Although both *CLIPA2* and *CLIPA14* kd mosquitoes exhibited a higher PO activity than *LacZ* kd control, this activity was significantly higher (2-fold; *p* = 0.0107) in *CLIPA14* relative to *CLIPA2* kd (Fig. 5*B*), which correlates well with the increased parasite melanization observed in the former genotype. Interestingly, the PO activity in *CLIPA14*/*CLIPA2* dkd was even higher than that in *CLIPA14* single kd, revealing an additive effect when both genes were cosilenced. Altogether, our results suggest that CLIPA2 and CLIPA14 act concertedly to regulate the TEP1-mediated immune response leading to melanization.

### CLIPA14 steady-state protein levels are not influenced by TEP1

**Figure 6.**
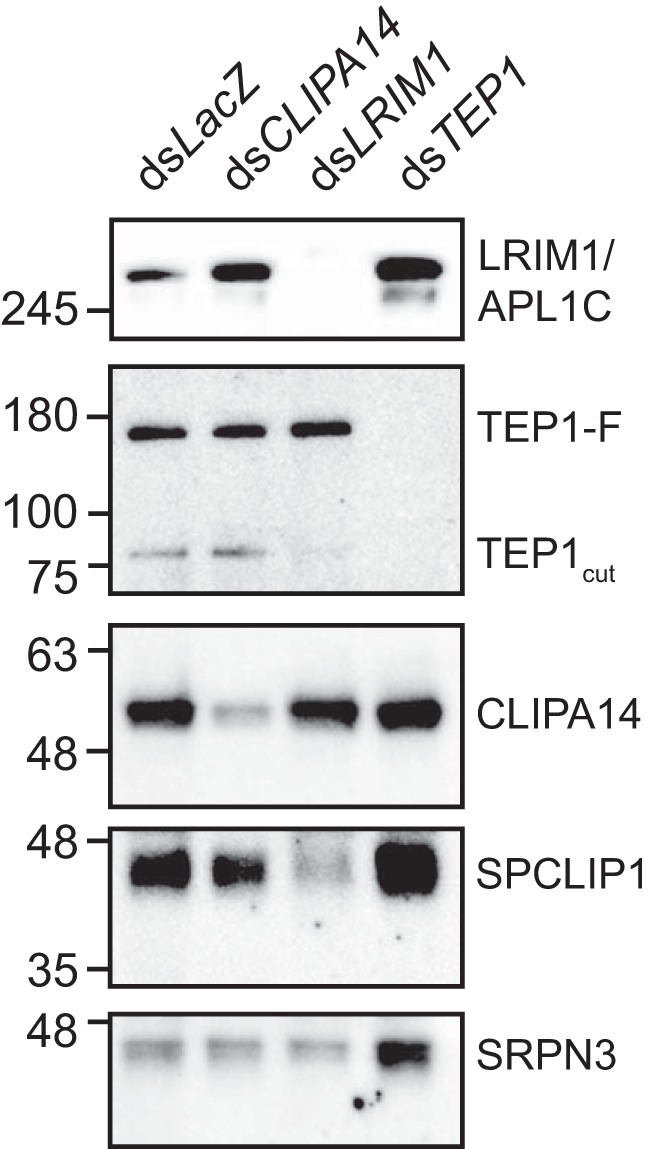
**CLIPA14 steady-state levels are not influenced by TEP1.** A Western blot analysis of hemolymph extracts collected 2 days after injecting naïve mosquitoes with the indicated dsRNAs is shown. The membrane was probed with antibodies against CLIPA14, TEP1, LRIM1, SPCLIP1, and SRPN3 (as a loading control). Protein quantification was performed using the Bradford protein assay, and 0.9 μg of hemolymph proteins was loaded per lane. The image is representative of two independent biological experiments.

TEP1_cut_ is stabilized in the mosquito hemolymph by LRIM1 and APL1C, and silencing either *LRIM1* or *APL1C* in naïve mosquitoes triggers the loss of TEP1_cut_ from the hemolymph (31, 32). We have previously shown that in *LRIM1* kd naïve mosquitoes SPCLIP1 (23) and CLIPA2 (30) are almost lost from the hemolymph concomitant with the loss of TEP1_cut_, suggesting that the steady-state hemolymph levels of these proteins are directly dependent on TEP1_cut_. To determine whether a similar correlation exists between CLIPA14 and TEP1, naïve mosquitoes were injected with dsRNAs corresponding to *LacZ* (control), *CLIPA14*, *TEP1*, and *LRIM1*, and hemolymph was extracted 48 h later for Western blot analysis. As reported previously, *LRIM1* kd triggered a strong reduction in TEP1_cut_ and SPCLIP1 levels in the hemolymph; however, no effect was observed on CLIPA14, suggesting that CLIPA14 steady-state levels are not regulated by TEP1_cut_ (Fig. 6).

**Figure 7.**
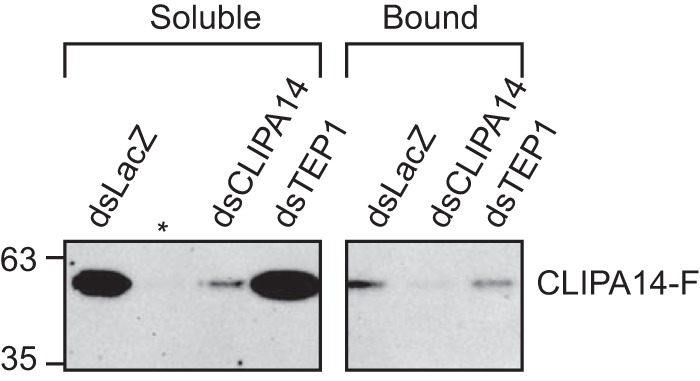
**CLIPA14 localizes to *E. coli* bioparticles in a TEP1-independent manner.** Hemolymph containing *E. coli* bioparticles was extracted from the indicated mosquito genotypes 15 min after bioparticle inoculation into the hemocoel. Bioparticles were pelleted by centrifugation, and the soluble fractions were collected. Bacterial pellets were washed with PBS, and bound proteins were extracted with protein loading buffer. Shown is a Western blot analysis of the soluble and bound fractions. The image is representative of two independent experiments. The *asterisk* indicates an empty lane.

To further investigate the nature of the relationship between CLIPA14 and TEP1, we asked whether CLIPA14 follows TEP1 to bacterial surfaces using the previously described *E. coli* bioparticle surface extraction assay (23, 30). Using this assay, we had shown previously that CLIPA2 (30) and SPCLIP1 (23) are recruited to bioparticle surfaces in a TEP1-dependent manner, suggesting that SPHs may act locally to modulate immune responses on microbial surfaces. As shown in Fig. 7, a small fraction of CLIPA14 did bind to bioparticle surfaces in control (ds*LacZ*) mosquitoes; however, *TEP1* kd did not abolish this binding, suggesting that TEP1 does not mediate CLIPA14 localization to bacterial surfaces. It is worth noting that, compared with CLIPA14, SPCLIP1 and CLIPA2 recruitment to bioparticles was more pronounced (23, 30), probably reflecting their strong dependence on TEP1_cut_.

## Discussion

The roles of clip domain-containing SPHs characterized in insects (15–17) and crustaceans (46) have been almost exclusively restricted to the positive regulation of the PPO cascade where they seem to act as cofactors for the proper cleavage and activation of PPO by PAPs. Although the mosquito CLIPA8 (22) is thought to fall within this functional category of SPHs, CLIPA2 (30) negatively regulates PPO activation indirectly by fine-tuning the level of activation of TEP1 in a yet undefined mechanism (7, 24, 26, 47). Here, we identify CLIPA14 as a novel key negative regulator of TEP1-mediated immune responses. *CLIPA14* and *CLIPA2* exhibit distinct RNAi phenotypes but also distinct relationships with TEP1. First, more parasites are melanized in *CLIPA14* (86%) compared with *CLIPA2* (56%; Ref. 30) kd mosquitoes. Second, although *CLIPA2* kd mosquitoes exhibited significantly enhanced tolerance to *E. coli* infections (30), *CLIPA14* kd mosquitoes showed basal level tolerance to *E. coli* but reduced tolerance to *S. aureus* infections. Third, the hemolymph PO activity in *CLIPA14* was 2-fold higher than that in *CLIPA2* kd mosquitoes after *E. coli* infections. However, silencing both genes exhibited an additive effect on PO activity, indicating that CLIPA2 and CLIPA14 cooperate to control the melanization response. In addition to differences in their RNAi phenotypes, CLIPA2 (30) and CLIPA14 steady-state levels in the hemolymph showed distinct control by TEP1_cut_. Additionally, CLIPA14 localization to bacterial surfaces does not seem to require TEP1 in contrast to that of CLIPA2, which is strictly TEP1-dependent (30). This indicates that CLIPA14 may be recruited to bacterial surfaces by a yet unknown PRR, or it may exhibit intrinsic ability to interact with microbial surfaces. In fact, some SPHs were reported to interact intrinsically with bacterial cell wall components (48, 49). Because the CLIPA14 binding pattern to bioparticles was weaker than that previously reported for CLIPA2 (30) and SPCLIP1 (23), we could not detect whether the cleaved CLIPA14-C is also bound to the bioparticles because this form is weakly detected in hemolymph extracts relative to the non-cleaved CLIPA14-F as shown in Fig. 4. *In vitro* binding assays whereby full-length and artificially cleaved recombinant CLIPA14 are incubated with bacteria will be required to provide a definitive answer as to the intrinsic ability of CLIPA14 to recognize bacterial cell wall components. *CLIPA14* kd mosquitoes are consistently resistant to bacterial infections as bacterial loads in tissues are reduced for all the species used in this study. However, tolerance varied depending on the bacterial species. Although no change in tolerance to *E. coli* was observed, *CLIPA14* kd reduced mosquito tolerance to *S. aureus* infections and slightly enhanced tolerance to *S. marcescens*. Although several factors influence tolerance such as wound repair mechanisms and the level of the immune response, we cannot exclude that the bacterial species used in this study may attain different loads in the tissues, which may influence the tolerance phenotype. This cannot be inferred from our cfu data because they were scored at one time point. Hence, a more dynamic assessment of cfu over a period of time may help explain the different tolerance phenotypes observed.

CLIPA14 infection-induced cleavage is clearly dependent on TEP1 and its positive regulator SPCLIP1, which further supports our previous observation that SPCLIP1 acts upstream in the TEP1 pathway whereby it seems to regulate the amount of active TEP1 that is deposited on microbial surfaces (23). The enhanced killing of *P. berghei* ookinetes and rapid clearance of *E. coli* in *CLIPA14* kd mosquitoes were clearly dependent on TEP1 function, suggesting that *CLIPA14* kd is triggering a potent TEP1-mediated response. How and at what level CLIPA14 negatively regulates the TEP1 response remain to be elucidated. We have previously shown that *CLIPA2* kd enhanced TEP1-F consumption during systemic infections, indicating that it is an upstream negative regulator of TEP1 (30); however, TEP1-F dynamics in *CLIPA14* kd mosquitoes did not provide convincing evidence to support a similar role for CLIPA14 (data not shown), suggesting that it may be acting downstream of CLIPA2.

Four SPHs have been involved so far in regulating mosquito immune responses; SPCLIP1 (23) and CLIPA8 (22) act as positive regulators, whereas CLIPA2 (30) and CLIPA14 act as negative regulators. The common feature among all four is that their functions are tightly linked to TEP1. It is intriguing that SPHs, despite being non-catalytic, have so far produced the most pronounced RNAi phenotypes with respect to *Plasmodium* ookinete survival among the larger mosquito clip domain family, which includes several catalytic members of the CLIPB and CLIPC subgroups. For instance, although CLIPA8 (21) and SPCLIP1 (23) kds completely abolished ookinete melanization in melanotic mosquito genotypes, the kd of several CLIPB genes showed only partial rescue (21). This suggests that SPHs play unique roles in mosquito immunity, whereas catalytic CLIPs may exhibit partial functional redundancy. The exact functions of these SPHs remain unknown, but their RNAi phenotypes suggest a multilayered regulation of the mosquito melanization response, unlike other insect SPHs whose role has been so far restricted to inducing the proper activation cleavage of PPO (16, 18, 45). It is tempting to speculate that SPHs may dictate the substrate specificity for certain catalytic CLIPs not only with respect to PPO but also to other downstream CLIPs in the cascade. The future characterization of the infection-induced cleavage patterns of catalytic CLIPs *in vivo* will be required to determine whether SPHs do act upstream of certain catalytic CLIPs. This will also facilitate the design of *in vitro* reconstitution assays that would gauge the effect of SPHs on the enzymatic activities and target specificities of candidate catalytic CLIPs. Conversely, the nature of the enzymes that cleave CLIPA14 and other mosquito SPHs remain unknown. By analogy to biochemical studies in other insects, we speculate that they belong to the catalytic CLIPB subgroup. Systematic RNAi screens of catalytic CLIPs are currently being conducted in our laboratory to identify candidate CLIPs required for the cleavage of CLIPA14 and other SPHs that exhibit clear infection-induced cleavage patterns such as CLIPA8.

Collectively, our results reveal so far an unprecedented complexity in the function of insect SPHs that extends beyond their commonly ascribed role as regulators of PPO activation cleavage. Future biochemical and biophysical studies will be required to highlight the exact molecular functions of these SPHs in CLIP cascades.

## Experimental procedures

### Ethical statement

This study was carried according to the recommendations in the Guide for the Care and Use of Laboratory Animals of the National Institutes of Health (Bethesda, MD). The animal protocol was approved by the Institutional Animal Care and Use Committee of the American University of Beirut (permit number 16-03-369). The Institutional Animal Care and Use Committee functions in compliance with the Public Health Service Policy on the Humane Care and Use of Laboratory Animals (United States) and adopts the Guide for the Care and Use of Laboratory Animals of the National Institutes of Health.

### A. gambiae rearing and infections with P. berghei

Experiments were performed with *A. gambiae* G3 strain. Mosquitoes were maintained at 27 ± 0.5 °C and 80 ± 5% humidity with a 12-h day–night cycle. Larvae were reared on Tetra Goldfish food, and freshly emerged mosquitoes were collected from larval pans using a vacuum collector. Adult mosquitoes were maintained on 10% sucrose and given BALB/c mouse blood (mice were anesthetized with ketamine) for egg production. *P. berghei* (PbGFPCON) constitutively expressing GFP (50) was propagated in 5–6-week-old BALB/c mice from frozen stocks. Parasite transmission to mosquitoes was performed by feeding on anesthetized mice containing a blood parasitemia of 4–6% for ∼20 min at 20 °C. Mosquitoes were then maintained on a 10% sucrose solution at 20 °C until processing. Mosquito midguts were dissected 7–8 days after feeding on *Plasmodium*-infected blood, and parasite counts were performed as described previously (21). The results shown are from three independent biological experiments. The Mann–Whitney log-rank test was used to calculate statistical significance, and medians were considered significantly different if *p* was <0.05.

### Synthesis of dsRNA for RNA interference

dsRNAs for *LacZ* (control) (51), *TEP1* (24), *CLIPA2* (30), and *LRIM1* (51) were synthesized using the previously reported T7 primers. dsRNA for *CLIPA14* was produced using the following pairs of T7-tagged primers (T7 tag underlined): T7-CLIPA14-F, 5′-TAATACGACTCACTATAGGGCGGCATCATCGACATCCGTGTC-3′ and T7-CLIPA14-R, 5′-TAATACGACTCACTATAGGGGTTGCTGTCGGCGACACGCTCCT-3′. T7-tagged *CLIPA14* amplicons are 326 bp in size. We have previously reported the synthesis of *CLIPA5* dsRNA (21), which as we showed here cross-silences *CLIPA14*. Hence, we redesigned a new *CLIPA5*-specific dsRNA using the T7-tagged primers (T7 tag underlined): T7-CLIPA5-F, 5′-TAATACGACTCACTATAGGGATTCGAGTTAATGCTGAACCTGA-3′ and T7-CLIPA5-R, 5′-TAATACGACTCACTATAGGGTGTCCATTGGACTTGATAGCATT-3′. The size of the T7-tagged CLIPA5 amplicon is 287 bp. T7-tagged amplicons were purified with GFX PCR DNA and Gel Band purification kit (GE Healthcare) and used as templates for dsRNA synthesis using the TranscriptAid T7 High Yield Transcription kit (Fermentas). Gene silencing was performed by injecting 69 nl of a 3 μg/μl solution of gene-specific dsRNA into the abdomen of each mosquito as described previously (52). To perform double-gene knockdowns, 138 nl of a solution containing a mixture of two dsRNAs, each at a concentration of 3 μg/μl, was used to inject mosquitoes.

### Mosquito survival assays and bacterial cfu counts

Mosquito bacterial infections were performed by injecting dsRNA-treated mosquitoes with ampicillin-resistant *E. coli* strain OP-50 (53) and tetracycline-resistant *S. aureus* (22) at an *A*_600 nm_ of 0.4 or by feeding mosquitoes on sugar pads containing DsRed-expressing gentamycin-resistant *S. marcescens* strain DB11 (43) at an *A*_600 nm_ of 0.1. Concerning the latter route of infection, mosquitoes were allowed to feed on sugar pads containing *S. marcescens* and a red food colorant for 2 days after which non-fed mosquitoes (lacking red color in abdomen) were sorted out under a stereoscope, whereas the rest were returned back to the cups and provided with sugar pads that do not contain *Serratia*, and their survival was scored thereafter. A batch of at least 40 adult female mosquitoes was infected per genotype. Mosquito survival rates were scored on a daily basis over 9 days. The Kaplan–Meier survival test was used to calculate percent survival. Statistical significance of the observed differences was calculated using the log-rank test. Survival assays were repeated at least three times. cfu counts for *E. coli* and *S. aureus* were performed exactly as described previously (30). Because CLIPA14 is a hemolymph protein involved in regulating systemic responses, we opted to measure the cfu levels of *Serratia* in the hemocoel rather than in whole mosquitoes to eliminate bias from gut-resident *Serratia* that are subject to distinct immune control. To that end, each *Serratia*-infected mosquito was dissected in a 30-μl drop of sterile PBS to remove the gut, which was discarded. The remaining carcass in addition to the PBS drop were transferred to a 1.5-ml Eppendorf tube. Carcasses were ground in batches of eight, and lysates were plated in serial dilutions on agar plates containing gentamycin to score for *Serratia* cfu. All bacterial cfu assays were performed three times, and statistical significance was calculated using the Mann–Whitney log-rank test. Medians were considered significantly different if *p* was <0.05.

### Expression of CLIPA14 in Sf9 cells and generation of CLIPA14 antibodies

The entire *CLIPA14* open reading frames lacking the endogenous signal peptide were amplified from adult mosquito cDNA using the following primer pair: CLIPA14-LIC-F, GACGACGACAAGATGCAG GATACGCTCGACGACCTC and CLIPA14-LIC-R, GAGGAGAAGCCCGGTTTCGGCGTGTAGACATAGTCCCG. The underlined sequences are extensions to allow ligase-independent cloning. The amplicons were cloned into pIEx^TM^-10 plasmid (Novagen) as a fusion with an N-terminal streptavidin tag and a C-terminal His tag coding for 10 histidines using a ligase-independent cloning (LIC) kit (Novagen) according to the manufacturer's protocol and sequenced. Because the predicted CLIPA14 coding sequence in VectorBase is partial, the sequenced full-length CLIPA14 coding sequence was deposited in GenBank^TM^ under accession number KY344791. Production of Sf9 cells that stably express CLIPA14^His^ and the purification of recombinant proteins from conditioned medium were performed as described previously (30). Purified recombinant CLIPA14^His^ protein was used to generate a rabbit polyclonal antibody (Eurogentec). CLIPA14 antibody was affinity-purified over an AminoLink column (Pierce) containing covalently bound CLIPA14^His^ according to the manufacturer's protocol.

### Western blot analysis

To determine the infection-induced cleavage of CLIPA14, hemolymph was extracted from ds*LacZ* (control)-, ds*CLIPA14*-, ds*TEP1*-, and ds*SPCLIP1*-treated mosquitoes at 1 h after intrathoracic injection of *S. aureus* (*A*_600 nm_ = 0.8). Wild-type naïve mosquitoes were used as a control. Hemolymph protein quantification was performed using the Bradford protein assay (Fermentas), and 1.2 μg of proteins were loaded per well for all samples in a given experiment. Proteins were resolved by reducing SDS-PAGE and transferred to Immun-Blot PVDF membrane (Bio-Rad) using wet transfer (Bio-Rad). Blots were incubated with rabbit αCLIPA14 and rabbit αSRPN3 (as a loading control; a kind gift from Kristin Michel, Kansas State University) at dilutions of 1:2500 and 1:1000, respectively. Anti-rabbit IgG horseradish peroxidase-conjugated secondary antibody (Abcam) was used at 1:12,000. The experiment was repeated there times with similar results.

To study the effects of *LRIM1* kd on CLIPA14 steady-state protein levels, hemolymph was extracted from naïve mosquitoes 48 h post-dsRNA injection. Hemolymph proteins were resolved by non-reducing SDS-PAGE, and Western blotting was performed as described above. The membrane was probed with rabbit αCLIPA14, rabbit αTEP1, rabbit αAPL1C (34), rabbit αSPCLIP1 (23), and rabbit αSRPN3 (as a loading control) at dilutions of 1:2500, 1:1000, 1:2000, 1:1000, and 1:1000, respectively. Anti-rabbit IgG horseradish peroxidase-conjugated secondary antibody was used at 1:12,000.

The localization of CLIPA14 to *E. coli* bioparticles was performed using bioparticle surface extraction exactly as described previously (23). Proteins in the soluble and bound fractions were resolved by non-reducing SDS-PAGE, and Western blotting was performed as described above. Blots were probed with rabbit αCLIPA14 (1:2500). The specificity of αCLIPA14 was determined as follows. Hemolymph was extracted 4 days postinjection of naïve mosquitoes with the indicated ds*CLIPA14*, ds*CLIPA5*, and ds*LacZ* (control) and subjected to Western blot analysis. The dilutions of αCLIPA14 primary and anti-rabbit IgG horseradish peroxidase-conjugated secondary antibodies were as indicated above. Efficient silencing of CLIPA2 was determined by Western blotting hemolymph extracted from naïve mosquitoes 4 days after ds*CLIPA2* injection. αCLIPA2 was used at 1:1000 as described previously (30).

### Phenoloxidase enzymatic assay

The PO enzymatic assay was performed 3 h after mosquito injection with *E. coli* strain OP-50 (*A*_600 nm_ = 0.8) using ∼5–9 μg of mosquito hemolymph per reaction as described previously (22). The absorbance at 492 nm was measured 30 min after incubation with l-3,4-dihydroxyphenylalanine (l-DOPA) (Sigma) in a Multiskan Ex microplate reader (Thermo Labsystems). The experiment was repeated three times using different batches of mosquitoes and *E. coli* cultures.

## Author contributions

M. A. O., N. J., and G. K. C. conceived and designed the experiments. J. N. performed the experiments. M. A. O., J. N., and G. K. C. analyzed the data. M. A. O. wrote the paper. J. N. and G. K. C. critically reviewed the manuscript.

## Supplementary Material

Supplemental Data

## Footnotes

3 Please note that the JBC is not responsible for the long-term archiving and maintenance of this site or any other third party-hosted site.

## Footnotes

2 The abbreviations used are:

CLIP: clip domain serine protease
PRR: pattern recognition receptor
SPH: clip domain serine protease homolog
kd: knockdown
PPO: prophenoloxidase
PO: phenoloxidase
PAP: prophenoloxidase-activating protease
TEP1: thioester-containing protein 1
TEP1-F: full-length TEP1
TEP1_cut_: processed form of TEP1
ds: double-stranded
dkd: double kd
CLIPA14-F: full-length CLIPA14
CLIPA14-C: C-terminal domain of CLIPA14
LIC: ligase-independent cloning
l-DOPA: l-3,4-dihydroxyphenylalanine.
