# Supplementary material for: The serine protease homolog CLIPA14 modulates the intensity of the immune response in the mosquito Anopheles gambiae
Source: J Biol Chem. 2017 Sep 19;292(44):18217–26. doi: 10.1074/jbc.M117.797787 (PMC5672044; doi:10.1074/jbc.M117.797787)
Supplement: Supplemental Data [file supp_292_44_18217__index.html]

The serine protease homolog CLIPA14 modulates the intensity of the immune response in the mosquito Anopheles gambiae — The serine protease homolog CLIPA14 modulates the intensity of the immune response in the mosquito Anopheles gambiae — CLIPA14, a novel regulator of mosquito immune responses — Supplemental Data 

# The serine protease homolog CLIPA14 modulates the intensity of the immune response in the mosquito *Anopheles gambiae*

## Supplemental Data

- suuplemental data (.pdf, 1.1 MB) - Supplemental Data with changes in legend of FigS4 highlighted
