# Supplementary material for: The serine protease homolog CLIPA14 modulates the intensity of the immune response in the mosquito Anopheles gambiae
Source: J Biol Chem. 2017 Sep 19;292(44):18217–26. doi: 10.1074/jbc.M117.797787 (PMC5672044; doi:10.1074/jbc.M117.797787)
Supplement: Supplemental Data [file 10.1074_M117.797787_jbc.M117.797787-1.pdf]

The serine protease homolog CLIPA14 modulates the intensity of the immune response in the mosquito *Anopheles gambiae*

Johnny Nakhleh[1], George K. Christophides[2], Mike A. Osta[1]*

From the [1]Department of Biology, American University of Beirut, Beirut, Lebanon. [2]Department of Life Sciences, Imperial College London, London, SW7 2AZ, United Kingdom

Supplemental Figure S1
Supplemental Figure S2
Supplemental Figure S3
Supplemental Figure S4
Supplemental Figure S5
Supplemental Figure S6

```
CLIPA5          1 --------------------MRWQVGAFFILLVTFGD--------PAKGHAIKGIESDES 60
CLIPA14-full      MLSRTVAVLAAVALMAGLVASQDTLDDLYLSLYNLTENKNTVTEPPAPPPAPAA-AAAPA
AGAP011788        MLSRTVAVLAAVALMAGLVASQDTLDDLYLSLYNLTENKNTVTEPPAPPPAPAAAAAAPA
                                                 *             **   *

CLIPA5         61 WDIPNSNANTSPNASCTGECVPYYLCKDNKIIKNGRGVIDIRVNAEP----ECPHYLETC 120
CLIPA14-full      AAPAPAPAPAQPRYTCTGECVQYYLCSDNKIITDGAGIIDIRVGEDPAEEYECPHFLNTC
AGAP011788        AAPAPAPAPAQPRYTCTGECVQYYLCSDNKIITDGAGIIDIRVGEDPAEEYECPHFLNTC
                         *   *   ****** **** *****  * * *****   *    **** * **

CLIPA5        121 CNARSVLDSPPPGVIK--PSGRTEQVRPTCGVRNKNGLGFSVTGVKDGESHYGEFPWMVA 180
CLIPA14-full      CEKDSVLDEPPPSATKAPPTSVPDARRPTCGMRNENGIGFRIEGQKDGESEYGEFPWMLA
AGAP011788        CEKDSVLDEPPPSATKAPPTSVPDARRPTCGMRNENGIGFRIEGQKDGESEYGEFPWMLA
                  *   **** ***   *  *       ***** ** ** **   * ***** ******* *

CLIPA5        181 VMLSSPMDNSDSILNVYQCGGSVIAPNVVLTAAHCVFNKPKTQLLLRAGEWDTQTEHELY 240
CLIPA14-full      VLREERVADSN--LNVYECGASLIAPNVVLTAAHCVFNKQKEQLLIRAGEWDTQTRNELY
AGAP011788        VLREERVADSN--LNVYECGASLIAPNVVLTAAHCVFNKQKEQLLIRAGEWDTQTRNELY
                  *        *   **** ** * **************** * *** *********  ***

CLIPA5        241 MHQNRRVAEVILHEAFDNESLANDVALLTLAEPFQLGENVQPICLPPSGTSFDYQHCFAS 300
CLIPA14-full      QHQDRRVAEVITHEAFNKASLANDVALLILTEPFQLAENVQPICLPPKGTSFDRTKCFAS
AGAP011788        QHQDRRVAEVITHEAFNKASLANDVALLILTEPFQLAENVQPI-----------------
                   ** ******* ****   ********* * ***** ********** *****   ****

CLIPA5        301 GWGKDQFGKEGKYQVILKKVELPVVPHAKCQETMRSQRVGNWFVLDQSFLCAGGVAGQDM 360
CLIPA14-full      GWGKNVFGKEGKYQVILKKVELPVVPHTECQQSLRSTRLGKRFALHQSFLCAGGVAGKDT
AGAP011788        ------------------------------------------------------------
                  ****   ********************  **   ** * *  * * ***********

CLIPA5        361 CRGDGGSPLVCPIPGSPTHYYQAGIVAWGLGCGEDGIPGVYGDVAFLRDWIDQQLVENSI 420
CLIPA14-full      CRGDGGSPLVCPVPGSPTHYYQAGIVAWGIGCGENGIPGVYGNVAFFRDWIDQQLVQRSI
AGAP011788        -----------------------------------------------------------
                  ************ **************** **** ******* *** *********  **

CLIPA5        421 LARDYYTFQAQ 431
CLIPA14-full      LARDYVYTP--
AGAP011788        -----------
                  *****
```

Figure S1: Alignment of the protein sequences of CLIPA5 and CLIPA14. Full-length CLIPA14 (CLIPA14-full) of our *A. gambiae* G3 strain was aligned with CLIPA5 (AGAP011787) and CLIPA14 (AGAP011788) sequences available in VectorBase using MUSCLE sequence alignment tool. Note that the CLIPA14 protein sequence in VectorBase (AGAP011788) is missing the last 146 aa (residues 283-429) of the C-terminal domain.

```
CLIPA5          GAGCAGG------TCCGTCCGACCTGTGGTGTGCGCAATAAAAATGGGCTCGGTTTTAGC
CLIPA14-full    GTGCCGGATGCGCGCCGTCCGACCTGCGGTATGCGCAACGAGAACGGCATCGGCTTCCGC
AGAP011788      GTGCCGGATGCGCGCCGTCCGACCTGCGGTATGCGCAACGAGAACGGCATCGGCTTCCGC
                * ** **       ************ *** *******  * ** **  **** **  **

CLIPA5          GTGACCGGTGTCAAGGACGGTGAATCACATTACGGTGAGTTCCCTTGGATGGTGGCCGTA
CLIPA14-full    ATCGAGGGCCAGAAGGATGGCGAGTCCGAGTACGGCGAGTTCCCGTGGATGTTGGCCGTG
AGAP011788      ATCGAGGGCCAGAAGGATGGCGAGTCCGAGTACGGCGAGTTCCCGTGGATGTTGGCCGTG
                 *    **    ***** ** ** **  * ***** ******** ****** *******

CLIPA5          ATGC-----TATCAAGTCCAATGGACAATAGCGACAGTATCTTGAATGTATACCAGTGCG
CLIPA14-full    CTGCGGGAGGAGCGTGTC-----------GCCGACAGCAACCTGAACGTGTACGAGTGCG
AGAP011788      CTGCGGGAGGAGCGTGTC-----------GCCGACAGCAACCTGAACGTGTACGAGTGCG
                 ***      * *  ***             ****** * * **** ** *** ******

CLIPA5          GTGGTTCAGTGATCGCTCCGAACGTTGTCCTGACGGCGGCACACTGTGTGTTTAACAAGC
CLIPA14-full    GTGCGTCGCTGATCGCGCCGAACGTGGTACTGACCGCGGCCCACTGCGTGTTCAACAAGC
AGAP011788      GTGCGTCGCTGATCGCGCCGAACGTGGTGCTGACCGCGGCCCACTGCGTGTTCAACAAGC
                ***  **  ******* ******** ** ***** ***** ***** ***** *******

CLIPA5          CGAAAACGCAGCTGCTGCTTCGGGCCGGCGAATGGGACACACAAACAGAACATGAGCTAT
CLIPA14-full    AGAAGGAGCAGCTGCTGATACGGGCCGGCGAATGGGACACGCAGACGCGCAACGAGCTGT
AGAP011788      AGAAGGAGCAGCTGCTGATACGGGCCGGCGAATGGGACACGCAGACGCGCAACGAGCTGT
                 ***   ********** * ******************** ** **     * ***** *

CLIPA5          ACATGCATCAGAACCGTCGAGTGGCTGAGGTCATCTTACATGAGGCGTTTGACAACGAAT
CLIPA14-full    ACCAGCATCAGGATCGCCGGGTCGCCGAGGTCATCACGCACGAAGCGTTCAACAAGGCGT
AGAP011788      ACCAGCATCAGGATCGCCGGGTCGCTGAGGTCATCACGCACGAAGCGTTCAACAAGGCGT
                **  ******* * ** ** ** ** *********   ** ** *****  **** *  *

CLIPA5          CATTGGCGAACGATGTGGCACTGCTAACCCTCGCCGAGCCGTTCCAGCTGGGAGAAAACG
CLIPA14-full    CGCTGGCGAACGATGTGGCACTGCTGATACTCACCGAGCCGTTCCAGCTGGCCGAGAACG
AGAP011788      CGCTGGCGAACGATGTGGCGCTGCTGATACTCACCGAGCCGTTCCAGCTGGCCGAGAACG
                *  **************** ***** *  *** ******************  ** ****

CLIPA5          TGCAGCCGATCTGTCTGCCGCCGAGTGGAACATCGTTCGACTATCAGCACTGTTTTGCTT
CLIPA14-full    TGCAGCCGATCTGTCTGCCGCCGAAGGGGACGTCGTTCGATCGCACCAAGTGTTTCGCCT
AGAP011788      TGCAGCCGATC-------------------------------------------------
                ***********

CLIPA5          CCGGCTGGGGTAAGGATCAGTTTGGCAAGGAGGGCAAGTACCAGGTGATACTGAAGAAGG
CLIPA14-full    CCGGCTGGGGCAAGAACGTGTTTGGCAAGGAGGGCAAGTACCAGGTGATACTGAAGAAGG
AGAP011788      ------------------------------------------------------------

CLIPA5          TCGAGCTGCCGGTCGTACCGCACGCTAAATGTCAAGAGACAATGCGATCTCAACGGGTTG
CLIPA14-full    TCGAGCTGCCGGTGGTGCCGCACACCGAGTGCCAACAGTCACTGCGCAGCACGCGGCTGG
AGAP011788      ------------------------------------------------------------

CLIPA5          GCAATTGGTTTGTGCTGGACCAGAGCTTCCTGTGTGCCGGTGGCGTGGCCGGGCAGGATA
CLIPA14-full    GCAAGCGGTTCGCGCTGCACCAGAGCTTCCTGTGCGCTGGCGGTGTGGCTGGAAAGGACA
AGAP011788      ------------------------------------------------------------

CLIPA5          TGTGCCGTGGTGATGGAGGCTCTCCGCTGGTGTGCCCGATTCCGGGATCGCCCACCCACT
CLIPA14-full    CGTGCCGTGGTGATGGAGGCTCTCCGCTGGTGTGTCCGGTTCCGGGATCGCCCACCCACT
AGAP011788      ------------------------------------------------------------
```

Figure S2. Alignment of the coding sequences of CLIPA5 and CLIPA14 corresponding to the protease-like domain. The coding sequence (cds) corresponding to the protease-like domain of full-length CLIPA14 (CLIPA14-full) cloned from the G3 strain was aligned with the respective VectorBase sequences of CLIPA5 (AGAP011787) and CLIPA14 (AGAP011788) using MUSCLE sequence alignment tool. The

sequence complementary to the dsRNA that was previously used to silence CLIPA5 (20) is underlined in red. Highlighted in yellow is a contiguous sequence of 53 nucleotides within the ds*CLIPA5* complementary region that shares 100% identity with its corresponding sequence in *CLIPA14* cds.

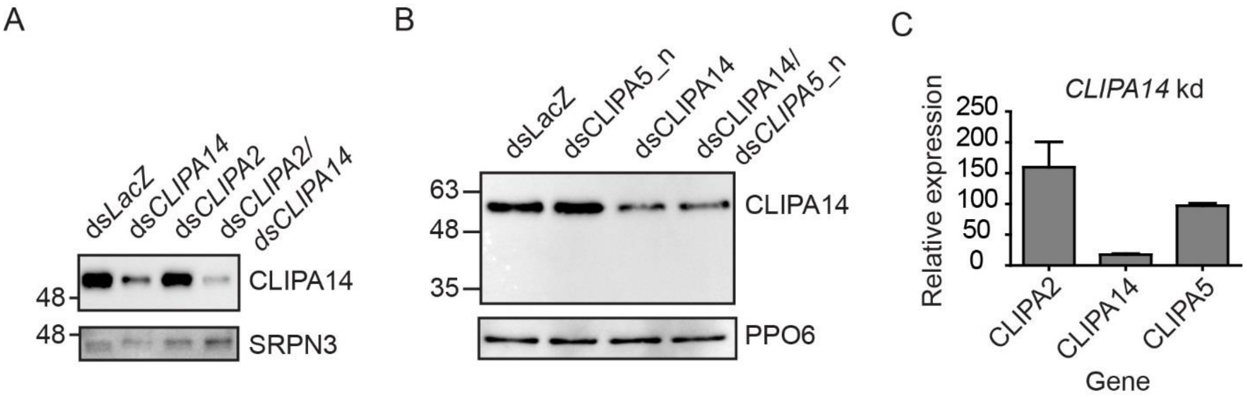

Figure S3. Absence of cross-silencing between the different CLIPAs. (A) Western blot showing the absence of cross-silencing between *CLIPA2* and *CLIPA14* in hemolymph extracts of mosquitoes treated with the indicated dsRNAs. The blot was probed with SRPN3 antibody to confirm equal loading. (B) Western blot showing the absence of cross-interaction between CLIPA14 antibody and CLIPA5. (C) *CLIPA14* kd does not alter the expression levels of CLIPA2 and CLIPA5. The transcript levels of *CLIPA2*, *CLIPA14* and *CLIPA5* were assessed by QRT-PCR in whole female mosquitoes four days following injection of *dsCLIPA14*. Error bars represent standard deviations in two representative biological experiments.

Figure S4

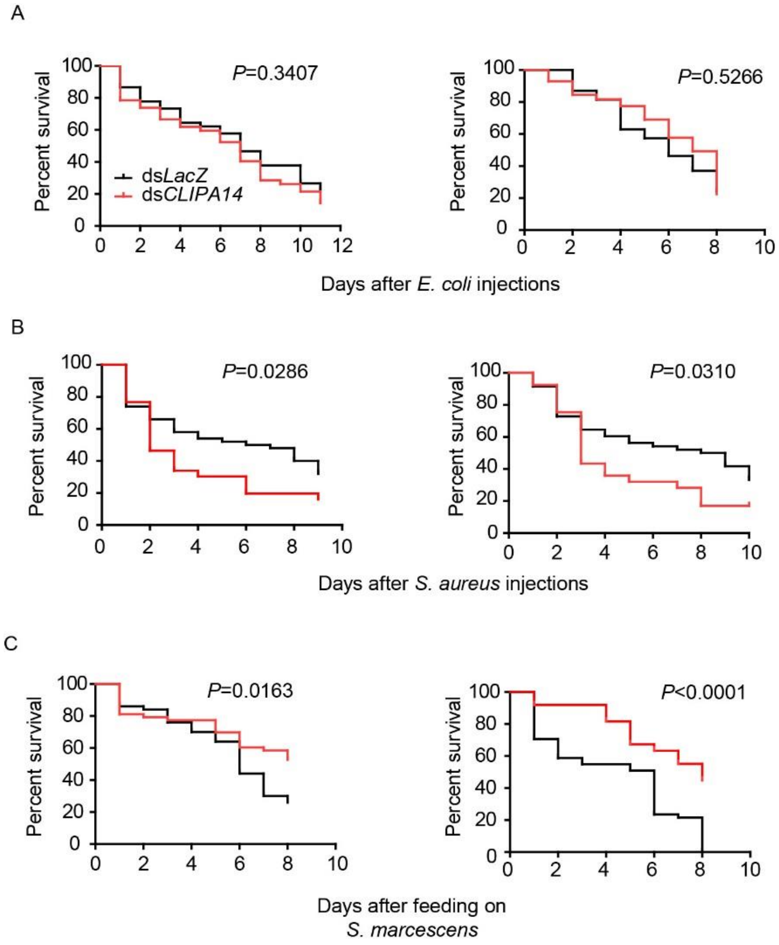

Figure S4. *CLIPA14* kd mosquitoes are resistant to bacterial infections. (A-C) mosquito survival assays following injection of (A) *E. coli* (OD_{600nm} = 0.4) and (B) *S. aureus* (OD_{600nm} = 0.4) into mosquito hemocoel, or (C) after oral infection with *S. marcescens*. Two representative experiments are shown from three independent biological experiments. The third replicate is shown in Fig 2. The Kaplan-Meier survival test was used to calculate the percent survival. Statistical significance of the observed differences was calculated using the Log-rank test.

Figure S5

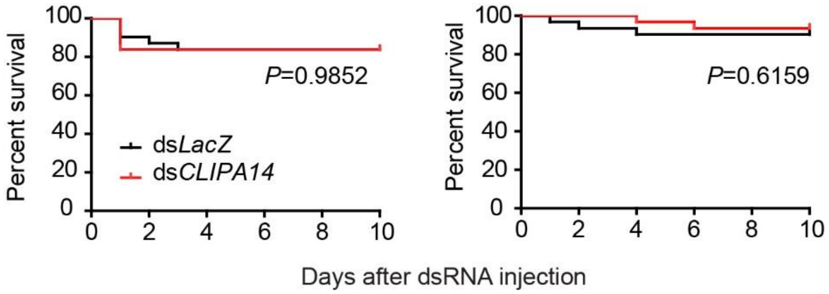

Figure S5. *CLIPA14* kd does not influence survival in naïve mosquitoes. Shown are two survival assays for naïve female mosquitoes injected with ds*CLIPA14* or ds*LacZ* (control). The Kaplan-Meier survival test was used to calculate the percent survival. Statistical significance of the observed differences was calculated using the Log-rank test.

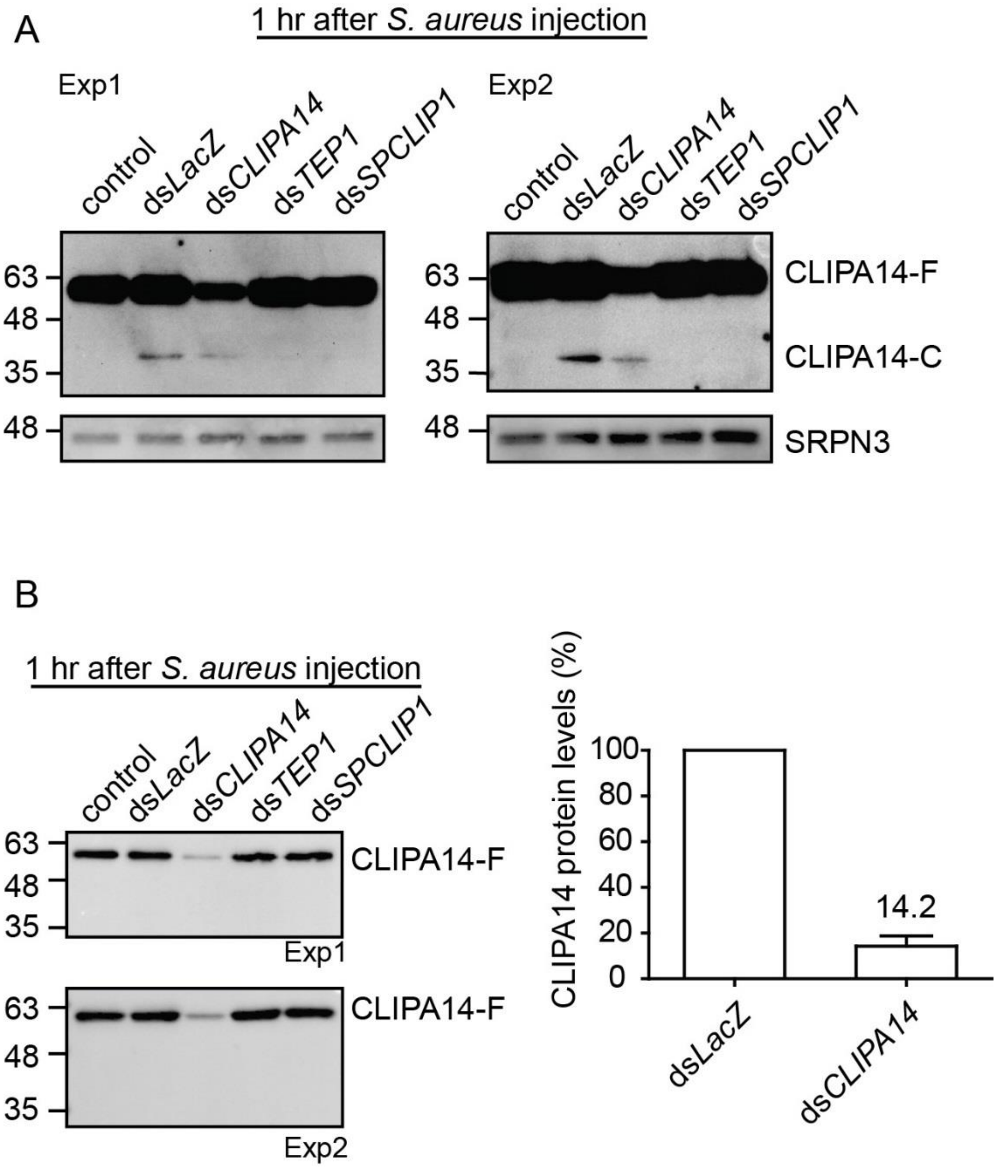

Figure S6. The infection-induced cleavage of CLIPA14 is dependent on TEP1 and SPCLIP1. (A) Western blots from independent biological experiments showing CLIPA14 cleavage in the indicated mosquito genotypes. Hemolymph was extracted from mosquitoes at 1 hr after injection with *S. aureus* (OD_{600}=0.8). Protein quantification was performed using the Bradford protein assay and 1.2 μg of hemolymph proteins were loaded per lane. The control group was not infected with *S. aureus*. The membranes were probed with antibodies against CLIPA14 and SRPN3 (as loading control). Shown are high exposure images (saturated) in order to detect CLIPA14-C cleaved form. (B) Low exposures (unsaturated) of the blots shown in (A). Note that at low exposures the cleaved form of CLIPA14 cannot be detected.

CLIPA14 protein levels in ds*CLIPA14* and ds*LacZ* mosquitoes were quantified from the two blots using ImageJ and presented in the bar graph.
